# Characterization of an orthovoltage biological irradiator used for radiobiological research

**DOI:** 10.1093/jrr/rru129

**Published:** 2015-02-17

**Authors:** Rezvan Azimi, Parham Alaei, Emiliano Spezi, Susanta K. Hui

**Affiliations:** 1 Department of Radiation Oncology, University of Minnesota, 420 Delaware Street, SE MMC 494, Minneapolis, MN 55455, USA; 2 Department of Medical Physics, Velindre Cancer Centre, Velindre Road, CF14 2TL, Cardiff, UK

**Keywords:** orthovoltage, dosimetry, Monte Carlo, biological irradiator

## Abstract

Orthovoltage irradiators are routinely used to irradiate specimens and small animals in biological research. There are several reports on the characteristics of these units for small field irradiations. However, there is limited knowledge about use of these units for large fields, which are essential for emerging large-field irregular shape irradiations, namely total marrow irradiation used as a conditioning regimen for hematological malignancies. This work describes characterization of a self-contained Orthovoltage biological irradiator for large fields using measurements and Monte Carlo simulations that could be used to compute the dose for *in vivo* or *in vitro* studies for large-field irradiation using this or a similar unit. Percentage depth dose, profiles, scatter factors, and half-value layers were measured and analyzed. A Monte Carlo model of the unit was created and used to generate depth dose and profiles, as well as scatter factors. An ion chamber array was also used for profile measurements of flatness and symmetry. The output was determined according to AAPM Task Group 61 guidelines. The depth dose measurements compare well with published data for similar beams. The Monte Carlo–generated depth dose and profiles match our measured doses to within 2%. Scatter factor measurements indicate gradual variation of these factors with field size. Dose rate measured by placing the ion chamber atop the unit's steel plate or solid water indicate enhanced readings of 5 to 28% compared with those measured in air. The stability of output over a 5-year period is within 2% of the 5-year average.

## INTRODUCTION

With the increasing utilization of small biological irradiators and microCT/PET scanners, cancer researchers are taking small animal radiation therapy research to new and advanced levels, similar to those employed in human radiation therapy. Hence, it is essential to properly characterize the radiation units used for animal research. The primary radiation delivery units used for this type of research are Orthovoltage ones.

A detailed review of small animal radiotherapy research platforms is provided by Verhaegen *et al.* [1]. Previous work in the characterization of Orthovoltage units includes that of Gerig *et al.* [2], Butson *et al.* [3], Aukett *et al.* [4], Evans *et al.* [5], and Verhaegen *et al.* [6] and was performed on older units primarily used for clinical treatments and employing applicators. While the first four references focused on measurement-based characterizations, Verhaegen *et al.* [6] developed computational models for two of these units (Philips MCN421 and Siemens Stabilipan 2 Th300) and tested the adequacy of two Monte Carlo codes (EGS4/BEAM and MCNP4B) in simulating the physics processes involved in the production of the X-ray beam.

More recently, Newton *et al.* [7] reported on the commissioning of the X-RAD 225cx unit (Precision X-Ray Inc., North Branford, CT) using radiochromic film and a novel 3D dosimetry system. Granton *et al.* [8] reported on a portal dosimetry and Monte Carlo–based method for evaluating the accuracy of treatment plans delivered by the same unit, which features 11 removable circular collimators and six rectangular collimators that can be individually attached to the machine. A recent paper by Lindsay *et al.* reported on the commissioning of the same unit in three institutions [9].

Furthermore, Pidikiti *et al.* [10] characterized the X-RAD 320 system (Precision X-Ray Inc., North Branford, CT). That work focused on the dosimetric characterization of the unit featuring six standard collimator apertures: 1.0, 2.0, 3.5, 5.0, 7.5 and 10.0 mm in diameter, with the aim of delivering small-field (focal) radiation for image-guided stereotactic small animal radiotherapy. An additional study by Aldelaijan *et al.* [11] used radiochromic film to measure the dose delivered at different depths in flasks irradiated using the X-RAD 320 unit. This work focused on characterizing the machine's output for the irradiation of blood specimens and was limited to one beam quality (320 kVp, with 2 mm of added Al filtration).

Quantitative accuracy of radiation delivery is important for large-field irradiation for *in vitro* studies, or large customized irregular- or regular-shaped fields for *in vivo* animal models, in which whole body or preferential locations of the body may be treated. Recently, total marrow irradiation (TMI) has emerged as a new conditioning regimen for hematological malignancies with goals to reduce relapse and toxicity [12, 13]. However, little is known about the impact of TMI on the bone marrow microenvironment, engraftment, and radiobiological aspects of this new treatment. Large-field mouse irradiation with appropriate compensators targeting radiation to a very large non-uniform skeletal system could potentially simulate TMI and serve as an excellent platform for basic research, emphasizing the need for focusing our attention on large-field irradiation.

The main goal of this investigation was the full dosimetric characterization of beams generated by the X-RAD 320 unit featuring an adjustable secondary collimator device for field sizes from 4 × 4 to up to 20 × 20 cm^2^. This has not been reported before for this unit. A second goal of the study was to develop and commission a Monte Carlo model of the X-RAD 320 unit for field sizes as large as 20 × 20 cm^2^ for further use in treatment planning and dose assessment. The beams characterized here are being used in the development of a model for TMI of small animals. The data presented here could be valuable for cancer researchers, who routinely use similar large-field beams in their radiation research work.

## MATERIALS AND METHODS

The X-RAD 320 Biological Irradiator is a self-contained X-ray system specifically used for the irradiation of small animals and biological specimens. The unit has an output voltage range of 5–320 kVp, a tube current range of 0.1–45 mA, and a maximum dose rate of 3 Gy/min at a 50 cm source-to-surface distance (SSD). The SSD is variable between 20 and 85 cm using an adjustable sample shelf made of steel. The beam shaping is done using a pair of 0.635-cm-thick lead collimators. The unit has a light field, similar to linear accelerators. The maximum field size is 20 × 20 cm^2^ at 50 cm. The X-rays are generated using an MXR-321 X-ray tube (Comet, Stamford, CT) powered by a GE high-voltage generator (General Electric, Fairfield, CT). The X-RAD 320 has no inherent filtration except for a 3-mm Beryllium window. A filter holder at the exit window allows beam conditioning filters to be added to achieve different beam qualities. Three filters: Thoraeus (0.8 mm Sn, 0.25 mm Cu, 1.5 mm Al), 2 mm Aluminum, and 0.35 mm Copper were used in this investigation, but the majority of beam data were collected using the 0.35-mm copper one.

### Dose rate measurements

The dose rate was measured according to guidelines set forth by the AAPM Task Group 61 [14], both in air and in phantom using a PTW 30012 0.6-cm^3^ farmer type ionization chamber (PTW, Hicksville, NY) calibrated with similar beam quality at an Accredited Dosimetry Calibration Laboratory (ADCL). For in-air measurements the chamber was secured in air at 50 cm source-to-chamber distance (SCD) using a clamp attached to a stand. In-phantom measurements were performed at 2-cm depth in solid water (Gammex457-CTG, Gammex RMI, Middleton, WI) with 6 cm of phantom as backscatter and an SSD of 50 cm. The AAPM TG-61 protocol specifies in-phantom measurements to be performed in liquid water and lists the determination of the dose to water using solid phantoms as an item for future consideration [14]. However, securing a water phantom that could fit in this unit and accurate positioning of an ionization chamber inside it is challenging as a routine output check task. The Institution of Physics and Engineering in Medicine and Biology (IPEMB) code of practice for the determination of absorbed dose for X-rays below 300 kV generating potential [15] allows the use of solid water for absorbed dose determination, provided an intercomparison with water is performed. We have made such an intercomparison between water and solid water in almost identical scatter conditions and determined the difference between water and solid water measurements to be <2%. In addition, using the Monte Carlo–generated beam spectrum at 2 cm depth in water and solid water, we calculated the mean mass energy absorption coefficients for the two phantoms using the NIST XCOM program [16] and found them to be within 0.3% of one another.

All the measurements in this study were performed with the collimator settings at their maximum position (20 × 20 cm^2^). This was repeated for three beam qualities/filter combinations: 320 kVp, 12.5 mAs, Thoraeus filter; 320 kVp, 12.5 mAs, 2 mm Al filter; and 225 kVp, 17 mAs, 0.35 mm Cu filter. The dose rate was also measured with the ion chamber resting on the unit's steel sample shelf and solid water slab (at 50 cm SCD) to compare various calibration conditions potentially employed and the effect of backscattered photons on the chamber readings. Placing the chamber on the steel or solid water positions its center at ∼0.5 cm from the surface. In order to make sure these measurements were made without disturbing the chamber, the chamber was fixed in place without the sample shelf, and the shelf was subsequently raised so that the chamber rested on it. The same method was repeated for solid water. Hence, an accurate comparison between measurements in air and with steel and solid water backscatter was made. The quantities used for dose rate measurements are summarized in Table 1. The equations to compute the dose rate in air and in phantom, adapted from the AAPM TG-61 protocol, are:

(1) $$D=M.N_{k}.P_{stem,air\cdot }{\left[{\left(\frac{\mu_{en}}{\rho }\right)}_{air}^{w}\right]}_{air}.$$

(2) $$D=M.N_{k}.P_{Q,Cham\cdot }{\left[{\left(\frac{\mu_{en}}{\rho }\right)}_{air}^{w}\right]}_{water}.$$

Equation 1 was used for in-air dose calculations and equation 2 for in-phantom ones. In these equations, M is the ion chamber reading, *N*_k_ is the air-kerma calibration factor, *P_stem,air_* is the chamber stem correction factor (taken as unity), *P_Q,cham_* is the chamber correction factor (listed in Table 1), and μ_*en*_/ρ is the mean mass energy absorption coefficient (listed in Table 1). Two factors used in the TG-61 protocol are not included above: the backscatter factor, which is material-dependent and was not used in the calculations, and the chamber waterproofing sleeve correction factor, which was not needed.

**Table 1.** Dosimetric factors used to compute the dose rate for different beam qualities in air and in the phantom according to Equations 1 and 2

kVp	225	320	320
mAs	17.0	12.5	12.5
Additional filtration	0.35 mmCu	2.0 mmAl	Thoraeus
${\left[{\left(\frac{\mu_{en}}{\rho }\right)}_{air}^{w}\right]}_{water}$	1.063	1.062	1.100
${\left[{\left(\frac{\mu_{en}}{\rho }\right)}_{air}^{w}\right]}_{air}$	1.078	1.076	1.106
*P_Q,Cham_*	1.021	1.021	1.006

All values extracted from AAPM TG-61 report [14].

### Half-value layer measurements

Half-value layers were determined using Copper sheets and the same ionization chamber secured in air for the three beam qualities stated above and using a narrow radiation beam of 2 × 2 cm^2^. The chamber was secured at 35 cm from the source housing in air, and an additional clamp was secured at 5 cm from the source housing for placement of Copper sheets. This is the best ‘good geometry’ setup achievable in this unit because of constraints due to the unit's enclosure and the presence of the sample shelf. First and second half-value layers were determined for the three beam qualities and compared with available nominal values.

### Scatter factor measurements

The in-air output ratio (S_c_), was measured at 50 cm SCD. Field sizes measured ranged from 2 × 2 to 20 × 20 cm^2^. The beam quality used for these measurements was 225 kVp, 17 mAs, 0.35 mm Cu filter. An Exradin A1SL miniature shonka plastic air equivalent thimble ionization chamber (Standard Imaging, Middleton, WI) was used for these measurements. The A1SL chamber has a collecting volume of 0.057 cm^3^. An average of three exposure readings were used and normalized to that of the 10 × 10 cm^2^ field.

The total scatter factors (S_c, p_) were measured in a solid water phantom with the chamber placed at 2-cm depth with 50-cm SSD. The same field sizes as those of the S_c_ measurements and the same A1SL chamber were used. The readings were similarly normalized to 10 × 10 cm^2^.

### Depth dose and profile measurements

Depth dose data were measured in solid water and water phantoms and also generated using Monte Carlo (MC) calculations for three field sizes: 4 × 4, 10 × 10 and 20 × 20 cm^2^ for depths ranging from 1 to 15 cm. The beam quality used for these measurements was 225 kVp, 17 mAs, 0.35 mm Cu filter. The differences in solid water and water materials for depth dose measurements have previously been investigated by Hill *et al.* [17] and were shown to agree within 1% in the kilovoltage energy range.

**Fig. 1.**
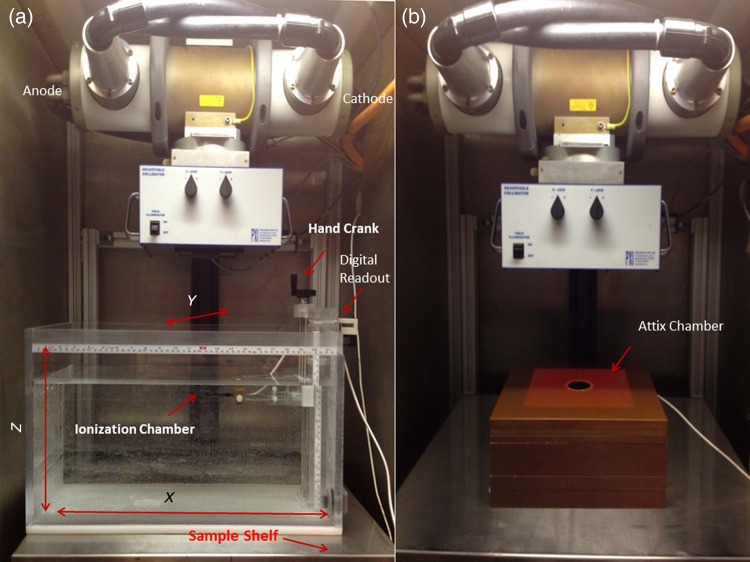
The water phantom used for depth dose and profile measurements (**a**) and the solid water phantom used for surface and buildup region depth dose measurements (**b**) inside the X-RAD 320 unit. The *x*-axis is along the anode–cathode direction, the *y-*axis is perpendicular to the anode–cathode direction, and the *z-*axis is along the beam line.

Figure 1a shows the water phantom used for measurements. As indicated in the figure, the anode–cathode direction is denoted as X and the opposite one as Y. The phantom is custom-made, with dimensions of 55 cm long by 30 cm wide by 30 cm high, made of Lucite. The A1SL ion chamber was held in a clamp that can be moved using hand-driven cranks with a calibrated measuring index to move the chamber in 0.1-cm increments. The depth dose measurements were taken at 1.0-cm increments in water. The positioning of the chamber is estimated to be accurate to <1 mm. An Attix parallel plate chamber (Gammex) was used for surface and near-surface measurements in a solid water phantom (Fig. 1b). Measurements from 0 to 1 cm depth were performed using this chamber in 0.2- and 0.3-cm increments, with the readings normalized to cylindrical chamber ones at 1-cm depth. Depth dose data was compared with the corresponding values published in the *British Journal of Radiology* (*BJR*) Supplement 25 [18].

The X profiles at 1, 5 and 10-cm depths were measured for the same field sizes in the water phantom and also generated using MC calculations. For profile measurements, an additional chamber holding apparatus was attached to the phantom, enabling moving the chamber along the long axis of the tank. For the X profiles, measurements were taken at every 0.5-cm increment within the field and at 0.2-cm increments in the penumbra region. For Y profiles, MC calculations and a commercial ion chamber array (IC Profiler, Sun Nuclear Corporation, Melbourne, FL) were used. The IC Profiler contains 251 ion chambers arranged in 0.5-cm spacing on X, Y and diagonal axes covering a field size of 32 × 32 cm^2^, with 0.9 cm of inherent water-equivalent buildup. Additional solid water buildup was used for measurements at 1, 5 and 10-cm depths. All profile data were normalized to corresponding depth dose values. The IC Profiler was calibrated for the 225 kVp, 17 mAs, 0.35 mm Cu filter beam prior to measurements, utilizing the largest field size and making sure all detectors were covered with the radiation beam. All IC Profiler measurements were made in continuous mode.

The beam symmetry and flatness were also evaluated using the profiler software for field sizes of 4 × 4, 10 × 10, and 20 × 20 cm^2^ at depths of 1, 5 and 10 cm. Equations 3 and 4 were used to determine flatness and symmetry:

(3) $$Flatness=\pm \frac{max-min}{max+min}.100,$$

in which ‘*max*’ is the maximum signal and ‘*min*’ is the minimum one within the central 80% of the field, determined dosimetrically.

(4) $$Symmetry=\frac{dose\;at\;point\;i-dose\;at\;point\;j}{dose\;at\;central\;axis\;in\;the\;same\;plane\;as\;points\;i\;and\;j}.100,$$

in which points ‘*i*’ and ‘*j*’ are any two points equidistant from the central axis within the central 80% of the field, determined dosimetrically.

### Monte Carlo simulation

**Fig. 2.**
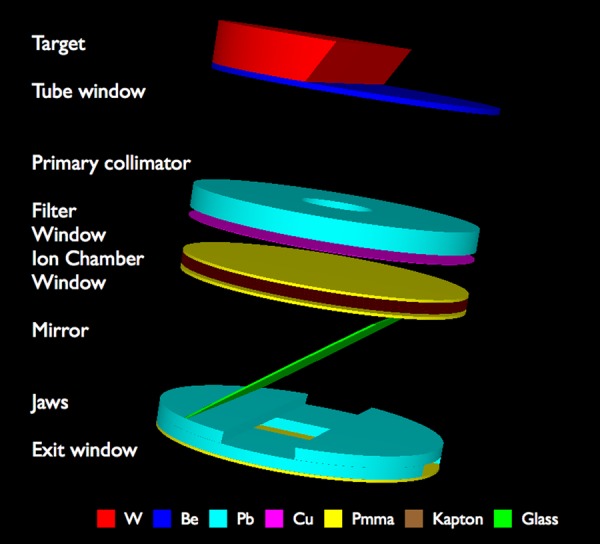
The schematic of the X-ray tube housing assembly used for Monte Carlo simulations.

A MC model of the X-RAD 320 Biological Irradiator was built using the BEAMnrc code version V4 2.3.2 [19], with construction details provided by the manufacturer. Figure 2 is a schematic of the X-ray tube housing used for Monte Carlo simulations built with the beampp package [20]. The MXR-321 X-ray tube was modeled with the XTUBE component module (CM). The Tungsten target angle was set to 30°, as per technical specifications. The tube beamline, including the lead primary collimator, the kapton layers of the monitor ionization chamber (PTW 7862) and the filters, was simulated with the CONESTACK CM. The glass mirror mounted at 45° incident to the X-ray beam was modeled with the MIRROR CM. The 0.635-cm-thick lead adjustable collimators and the 0.15-cm PMMA protective plates at both ends of the device were simulated with the JAWS CM. The electron source was modeled as a parallel circular beam incident from the side with a radius of 0.25 cm. We simulated a total of 1200 × 10^6^ 225-keV monoenergetic electrons impinging on the target, and a phase space file (PHSP) was generated at the exit plane of the unit for every X-ray beam considered in this work. The PHSPs contained between 7 × 10^6^ (4 × 4 cm^2^ field) and 150 × 10^6^ particles (20 × 20 cm^2^ field) and were used as the source of the water phantom irradiation.

Directional bremsstrahlung splitting (DBS) was used to improve the efficiency of the simulations. The DBS splitting field radius was set to 12.5 cm, and the Bremsstrahlung splitting number was set to 5000, for a SSD of 50 cm [21]. Rayleigh data were generated with the PEGS4 code system for all the materials used in this work. Photoelectron angular sampling and other MC transport settings such as Rayleigh scattering, atomic relaxations, spin effect, and electron impact ionization were also included in the simulations and set on as described in previous work [22]. Electron and photon cutoff energies (ECUT and PCUT) were set to 0.521 and 0.01 MeV, respectively, for the simulation of the X-ray beamline. For electrons, ECUT includes both kinetic and rest mass energy. The simulation of the water phantom irradiation was carried out with the DOSXYZnrc code using the HOWFARLESS option [23]. Also, in this case, ECUT and PCUT were set to 0.521 and 0.01 MeV, respectively. The dose to water was deposited in an isotropic grid of 0.25 × 0.25 × 0.25 cm^3^.

## RESULTS

### Dose rate measurements

The results of dose rate measurements are included in Table 2. The output measured atop the steel sample shelf and solid water indicates an increase of ∼5% and 20–28%, respectively, over that in air, as seen in the table. The stability of the output of the unit has been monitored for five years. An analysis of the data indicates the output of the unit to be stable and within 2% of the 5-year average. The dose rate data for the 225 kVp, 17 mA, 0.35 mm Cu beam is reported for both liquid and solid water phantoms, demonstrating their agreement to within 2%.

**Table 2.** Beam qualities, half values layers, and outputs for the three beams generated by the X-RAD 320 unit

kVp	225	320	320
mAs	17.0	12.5	12.5
Additional filtration	0.35 mmCu	2.0 mmAl	Thoraeus
Measured HVL (mmCu)	1.15	0.8	3.9
Nominal HVL (mmCu)	N/A	1.0	3.7
Homogeneity coefficient	0.34	0.25	0.46
Dose rate in air (cGy/min) (50 cm SCD) (not including backscatter factor)	109	229	89
Dose rate at 2 cm depth in solid water phantom (cGy/min)	144	285	107
Dose rate at 2 cm depth in water phantom (cGy/min)	141.2		
Dose rate above sample shelf (cGy/min) (50 cm SCD)	115	241	94
Dose rate above solid water [cGy/min] (50 cm SCD)	140	289	107

All measurements performed with jaws fully open (field size of 20 × 20 cm^2^ at 50 cm). The dose rate for the primary beam investigated here (225 kVp, 17 mA, 0.35 mm Cu) was measured both in water and solid water, as explained in the manuscript.

### Half-value layer measurements

**Fig. 3.**
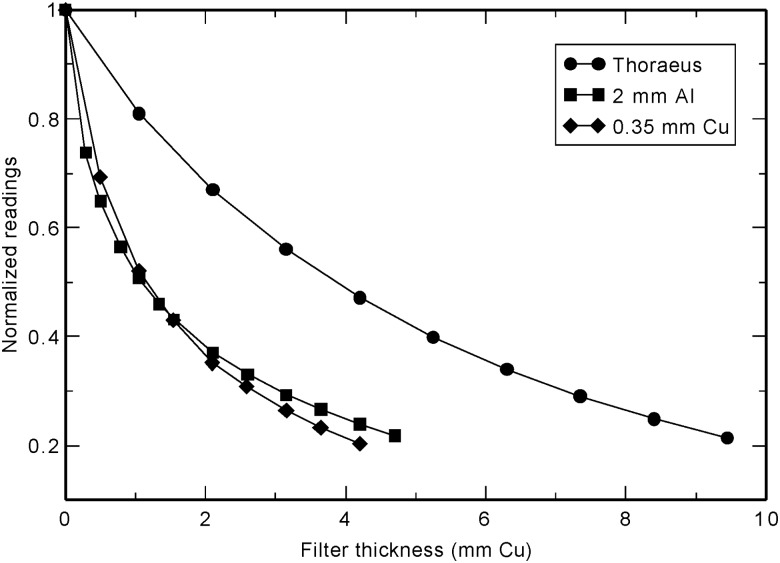
Half value layer measurement results for the three beam qualities: Thoraeus (320 kVp, 12.5 mA), 2 mm Al (320 kVp, 12.5 mA) and 0.35 mm Cu (225 kVp, 17 mA).

The results of half-value layer measurements are included in Table 2 and Fig. 3. Comparison of this data with nominal measurements shows slight differences.

### Scatter factor measurements

**Fig. 4.**
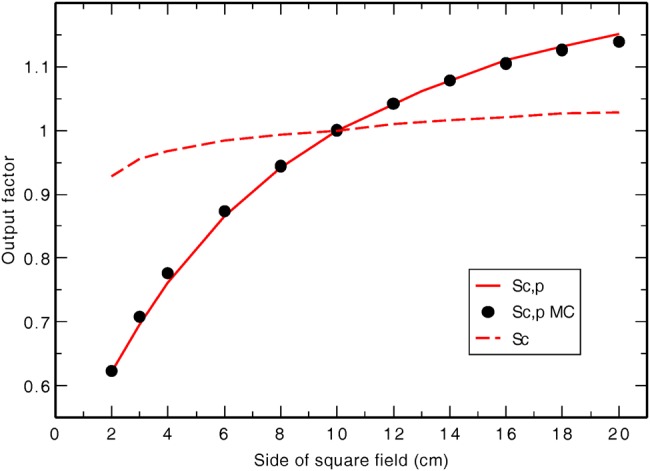
The measured (solid line) and Monte Carlo–generated (circles) in-phantom output factors [S_c,p_] and measured in-air output ratios, S_c_ (dashed line) as a function of field size for the 225-kVp, 17-mA, 0.35-mm Cu beam. The data are normalized to 10 × 10 cm^2^ field size values.

Figure 4 shows the variation of in-air output ratio (S_c_) and total scatter factor (S_c, p_) with field size. The output factors increase gradually with increase in field size, as expected. Monte Carlo–generated data are mostly within 1% of the experimental data, with a maximum deviation of 1.5% for the 4 × 4 cm^2^ field. This is within the statistical uncertainty of the calculations (between 1% and 2%, 1 standard deviation) and within measurement uncertainty estimated to be 2%.

### Depth dose and profile measurements

**Fig. 5.**
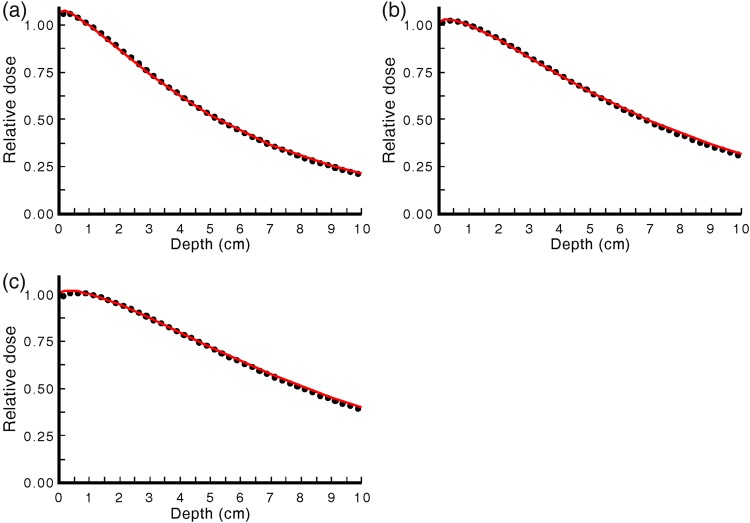
Depth dose values measured using an ionization chamber in water (line) and calculated using Monte Carlo (circle) for 4 × 4 (**a**), 10 × 10 (**b**) and 20 × 20 cm^2^ (**c**) for the 225-kVp, 17-mA, 0.35-mm Cu beam. The data is normalized to 1.0-cm depth.

Figure 5 shows measured and MC-generated depth dose curves for field sizes of 4 × 4, 10 × 10 and 20 × 20 cm^2^. Measurement uncertainty is estimated to be ± 2%, and the uncertainty of the MC datapoints is ± 2%. The MC calculations are in good agreement with measurements, with a maximum difference of 1% at the depth of 0.5 cm for 4 × 4 and 10 × 10 cm^2^ (Fig. 5a and b) and a difference of 2% for the 20 × 20 cm^2^ field (Fig. 5c). The agreement between measured and MC data beyond 1 cm is also very good, with a maximum deviation of 2% beyond 9 cm for the 20 × 20 cm^2^ field. Comparison of measured data with those published in *BJR* Supplement 25 for similar beam quality shows <2% difference.

**Fig. 6.**
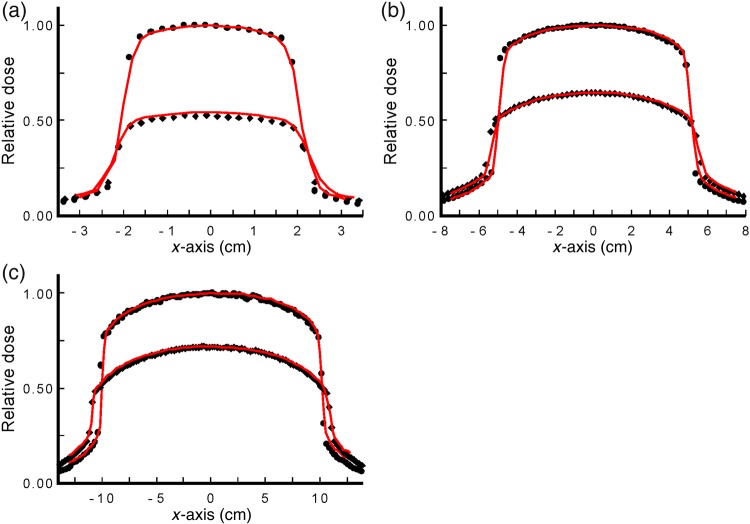
Profiles measured using the ionization chamber in water (line) and calculated using Monte Carlo (symbols) for 4 × 4 (**a**), 10 × 10 (**b**), and 20 × 20 cm^2^ (**c**) and depths of 1 cm and 5 cm (circle and diamond respectively) for the 2250-kVp, 17-mA, 0.35-mm Cu beam. The data is normalized to the central axis value at 1-cm depth.

Figure 6 shows X profiles at two depths of 1 and 5 cm for the same field sizes as above. As seen in the figure, there is an excellent agreement between the measured and calculated values within the field, with minor deviations observed in the penumbra and out-of-field regions. Symmetry at 1-cm depth for the 20 × 20 cm^2^ field size was 0.3% for the *y*-axis and 4.3% for the *x*-axis, and flatness was 6.0% and 5.1% for the *x* and *y* axes, respectively. The 10 × 10 cm^2^ field size exhibited the best flatness and symmetry among the fields studied.

## DISCUSSION

The full characterization of beams produced by the X-RAD 320 Orthovoltage unit featuring adjustable secondary lead collimators is described. To the best of our knowledge, this is the first time that this has been reported in such detail. We measured field sizes in the range of 4 × 4 to 20 × 20 cm^2^, and our data showed overall excellent correlation with Monte Carlo simulations. Measuring percent depth dose in near surface and in the buildup region is a challenging task, as stated in the AAPM TG 61 report [14]. Nevertheless, very good agreement between experiments and MC calculations was found in this region, with maximum discrepancies of only 2% for the largest field size. Due to the difficulty of accurately positioning the ion chamber in the *y* direction because of the unit's enclosure, no ion chamber profile measurements were performed in that direction. However, the accuracy of the IC Profiler measurements was tested by comparing the *x* profiles measured using an ion chamber in water with those measured using the device; hence its utility in measuring the profiles in the *y* direction. A comparison of *x* and *y* profiles measured by the IC Profiler showed them to be almost identical.

Output measurements in this energy range commonly follow the AAPM TG-61 protocol, specifying the measurements to be performed either in air or at a 2-cm depth in the phantom. Additional measurements were performed here by placing the chamber atop the steel sample plate or solid water because this has been employed as a more practical and reproducible set-up. Measurements atop the steel plate and solid water show an increase in dose of varying degrees, with steel plate ones enhancing the dose at a lower magnitude than solid water ones. So care must be exercised when using these values instead of the dose to air.

Another consideration regarding the output involves the collimation of the beam. As this unit is commonly calibrated with the maximum field size, collimating the beam to a small sample may result in gross overestimation of the dose. As seen in Fig. 4, the in-phantom output gradually increases with field size, as expected. Another observation made during this work concerned the limited useful area of the beam. This unit has movable jaws that can project to as large as 20 × 20 cm^2^ at 50 cm. This large field size may give the impression that the entire jaw-defined field could be used for uniform irradiation. Profile measurements, however, prove the contrary. Examination of Fig. 6 for the 20 × 20 cm^2^ field at 1-cm depth shows that the central 10-cm width of the field is covered by at least 95% of the dose at the central axis, whereas the central 14-cm width is covered by at least 90%; areas outside of this are covered by lower doses. So, if dose accuracy is of importance, care must be taken in utilizing the center part of the beam. The same applies for the smaller field sizes measured. This is, of course, influenced by the irradiation set-up and the phantom used. The presence of a heel effect affects the uniformity of the dose in the *x* direction slightly. This heel effect is only visible in the 1-cm depth profiles. There is a maximum dose difference of 1% along the *x*-profile for the 10 × 10 and 20 × 20 cm^2^ field sizes within ± 3 and ± 6 cm of the central axis, respectively. This matches observation made by Knoos *et al.* for a similar beam [24].

The depth dose data generated here were compared with the *BJR* Supplement 25 data, but it is not feasible to compare them to those of others. For example, Gerig *et al.* [2], Butson *et al.* [3], Aukett *et al.* [4] and Evans *et al.* [5] collected beam data on the DXT-300 and Gulmay D3300 Orthovoltage units, which had similar tubes to that of X-RAD 320 but different filtrations, and primarily utilized applicators as opposed to jaws. On the other hand, Newton *et al.* [7], Granton *et al.* [8] and Pidikiti *et al.* [10] assessed Orthovoltage beams produced by a similar or the same unit as the one in this study but focused on small field sizes and different collimation systems. Simulation of beam characteristics along with measurements facilitated benchmarking of the MC model for this system, which could further be used to predict the dose distribution within irradiated samples. Establishment of this method may offer quantitative measurements associating absolute radiation delivery and biological parameters in *in vivo* murine studies. Accurate measurement of beam data is a standard procedure for clinical machines that allow accurate patient treatment, and associate the dose delivered to clinical outcome. Murine studies are often used as qualitative studies in the absence of accurate dosimetry. Our current study is a step toward developing quantitative *in vivo* models to parallel clinical studies.

## FUNDING

This work was partially supported by the National Institutes of Health (grant number 1R01CA154491-01). This work was also partially supported by PHS Cancer Center Support Grant P30 CA77398 and seed grants from the University of Minnesota.
